# Engaging Assisted Living Residents in the Development of an Evaluation Plan for a Future Trial of an Exercise Program

**DOI:** 10.1093/geroni/igaf122.595

**Published:** 2025-12-31

**Authors:** Ann Reddy, Kevin Carlin, Olivia Taylor, Rosa Baier, Ellen McCreedy

**Affiliations:** Brown University, Providence, Rhode Island, United States; Meridian Senior Living, Bethesda, Maryland, United States; Brown University School of Public Health, Providence, Rhode Island, United States; Brown University School of Public Health, Providence, Rhode Island, United States; Brown University, School of Public Health, Providence, Rhode Island, United States

## Abstract

Many trials are conducted without first assessing which outcomes are important to patients receiving the interventions. The goal of this work was to ask participants of an ongoing exercise program what they believed the most important impacts of the program were to better align outcomes for a future trial with participant priorities. The Rev6 program is a seated, 30-minute exercise class designed for older adults with varying cognitive abilities. The program is offered twice a week by a large assisted living community (ALC) in Rhode Island. We observed three Rev6 sessions and held a focus group with seven Rev6 participants. We also interviewed one ALC staff member responsible for delivering the Rev6 program and two corporate leaders. Three members of the research team analyzed the focus group and interview transcripts to identify themes. Themes related to physical health included staff- and participant-reported impact of the Rev6 program on balance and walking, strength, and pain. Themes related to mental health included staff- and participant-reported impact of the Rev6 program on “feeling better” and increased social connection. With input from ALC staff, researchers developed visual cue cards for each theme and met with nine program participants to vote on the most important theme to evaluate as an outcome measure for a future study. Four residents voted for balance and walking, three residents voted for feeling better, and two residents voted for pain as the most important impact of the program. This framework for evaluation codesign includes the voices of people with dementia.

